# Manger space restriction does not negatively impact growth efficiency of feedlot heifers program fed a concentrate-based diet to gain 1.36 kg daily

**DOI:** 10.1093/tas/txad012

**Published:** 2023-01-27

**Authors:** Erin R Gubbels, Warren C Rusche, Zachary K Smith

**Affiliations:** Department of Animal Science, South Dakota State University, Brookings, SD 57007, USA; Department of Animal Science, South Dakota State University, Brookings, SD 57007, USA; Department of Animal Science, South Dakota State University, Brookings, SD 57007, USA

**Keywords:** Beef, bunk space, growing, growth, program feeding

## Abstract

The objective of this research was to determine the influence manger space restriction had on program-fed feedlot heifers during the growing phase. Charolais × Angus heifers [initial body weight (BW) = 329 ± 22.1 kg] were used in a 109-d backgrounding study. Heifers were received approximately 60 d prior to study initiation. Initial processing (53 d before study initiation) included individual BW, application of an identification tag, vaccination against viral respiratory pathogens and clostridial species, and administration of doramectin pour-on for control of internal and external parasites. All heifers were administered 36 mg of zeranol at study initiation and were assigned to 1 of 10 pens (*n* = 5 pens/treatment with 10 heifers/pen) in a randomized complete block design (blocked by location). Each pen was randomly assigned to 1 of 2 treatments: 20.3 cm (8IN) or 40.6 cm (16IN) of linear bunk space/heifer. Heifers were individually weighed on days 1, 14, 35, 63, 84, and 109. Heifers were programmed to gain 1.36 kg daily based on predictive equations set forth by the California Net Energy System. To calculate predictive values, a final BW of 575 kg was assumed to be the mature BW of the heifers and tabular net energy values of 2.05 NEm and 1.36 NEg from days 1 to 22, 2.00 NEm and 1.35 NEg from days 23 to 82, and 1.97 NEm and 1.32 NEg from days 83 to 109 were used. Data were analyzed using the GLIMMIX procedure of SAS 9.4 with manger space allocation as the fixed effect and block as the random effect. No differences (*P* > 0.35) were observed between 8IN or 16IN heifers for initial BW, final BW, average daily gain, dry matter intake, feed efficiency, variation in daily weight gain within each pen or applied energetic measures. No differences (*P* > 0.50) were observed between treatments for morbidity. Although not statistically analyzed, 8IN heifers appeared to have looser stools during the first 2 weeks compared to the 16IN heifers. These data suggest restricting manger space allocation from 40.6 to 20.3 cm did not negatively influence gain efficiency or the efficiency of dietary net energy utilization in heifers programmed fed a concentrate-based diet to gain 1.36 kg daily. The use of tabular net energy values and required net energy of maintenance and retained energy equations are effective means to program cattle to a desired rate of daily gain during the growing phase.

## Introduction

A main goal of a backgrounding program is to suppress fat or lipid deposition and promote the growth of bones and lean tissue through achieving less-than-maximal growth (Block et al., 2001). This is done in effort to prolong the growth curve of smaller-framed cattle to reach a more desirable mature weight when cattle are harvested at the end of the finishing period (Owens et al., 1993). Feed intake is known to be a direct correlation of beef cattle growth. Therefore, the management of feed intake plays an important role in prolonging the growth curve in cattle, particularly those in the background phase of production (Galyean et al., 1999).

Controlling or managing feed intake relative to the ad libitum amount is not a new concept to the cattle feeding industry (Galyean et al., 1999). Feed intake can be managed via restricted feeding or programmed feeding (Galyean et al., 1999). Limited or restricted feeding during the backgrounding phase would include management where feed intake is restricted relative to the actual or predicted ad libitum intake of a pen of cattle (Galyean et al., 1999). The restriction of dry matter intake (DMI) has led to improvements in feed efficiency (Hicks et al., 1990; Loerch and Fluharty, 1998) and ultimately resulted in reduced fat content of carcasses. Restricting the energy content of the diet via increased roughage inclusion or by limiting the amount of a high-concentrate diet that is fed has also been suggested to manage growth rate during the backgrounding phase (Blom et al., 2022).

Program-fed cattle have shown to be more feed efficient and have decreased the total feed required per animal, thus resulting in feed costs saving compared to ad libitum fed cattle (Loerch and Fluharty, 1998). Program feeding refers to the use of net energy (NE) equations to calculate the quantity of feed required for maintenance at a desired rate of gain (Galyean et al., 1999). The use of NE equations from the California Net Energy System (CNES) has been proven to be an effective means of predicting expected growth measures in feedlot cattle (Galyean et al., 1999; Gunter et al., 1996; Loerch and Fluharty, 1998; Zinn, 1989). In general, the premise of using the equations set forth by the CNES determines the amount of daily intake by calculating the amount of feed required for daily maintenance plus the feed required for daily gain. These have been shown to be the most effective when tabular dietary NE values for maintenance and gain and expected final body weight (BW) are known (Zinn, 1989). A few studies have evaluated the use NE equations to predict expected growth values in program fed cattle with limited manger space. Zinn (1989) was able to program feed intake to allow for a desired rate of gain with limited bunk space. Gunter et al., (1996) was also able to achieve the desired gain during the growing period with a restricted amount of bunk space. No negative performance influences were attributed to program-fed cattle with limited bunk space in either of these studies.

Cattle performance, welfare, and health are dependent upon bunk space and can be negatively impacted in cattle experiencing restriction (Harrison and Oltjen, 2021). This poses a concern for cattle that are not allowed the recommended amount of linear bunk space. It is recommended when cattle of 360 to 545 kg are fed twice daily, that 27.9 to 33.0 cm of linear bunk space is provided (FASS, 2020). Bunk space allotments of 24.3 to 63.5 cm of linear bunk space per animal were recommended when DMI was restricted (Duncan et al., 2022). Allotments of 15 to 45 cm bunk space per animal did not negatively influence growth performance measures in steers that were limit-fed during the receiving phase (Zinn, 1989). We hypothesized that restricting manger space and program-fed heifers would not negatively alter feedlot performance. The objective of this study was to determine the influence of manger space restriction on feedlot heifers program fed to gain 1.36 kg daily during the growing phase on growth performance and health measures.

## Materials and Methods

### Institutional Animal Care and Use Approval

This study was conducted at the Ruminant Nutrition Center in Brookings, SD between March and July of 2022. The animal care and handling procedures used in this study were approved by the South Dakota State University Animal Care and Use Committee (2202-006E).

### Heifer Management and Treatments

Charolais × Angus crossbred heifers (initial BW = 329 ± 22.1 kg) were used in a 109-d study. Heifers were procured from a local South Dakota ranch and received approximately 60 d prior to study initiation. Initial processing was conducted 53 d before the initiation of the present experiment and included individual BW measurement (scale readability 0.454 kg), application of a unique identification and electronic ear tag, vaccination against viral respiratory pathogens (Bovi-Shield Gold 5, Zoetis, Parsippany, NJ) and clostridial species (Ultrabac/Somubac 7, Zoetis) all heifers were administered pour-on dormectin (Dectomax, Zoetis) for control of internal and external parasites. All heifers were administered 36 mg of zeranol (Ralgro, Merck Animal Health, Madison, NJ) at study initiation.

Heifers were assigned to 1 of 10 uncovered pens (7.62 m × 7.62 m concrete surface pens with 7.62 m of concrete bunk; 5 pens/treatment; 10 heifers per pen) in a randomized complete block design (blocked by location) and pen was randomly assigned to 1 of 2 treatments: 20.3 linear cm of bunk space per heifer (8IN) or 40.6 linear cm of bunk space per heifer (16IN). Pen space and animal space were not confounded based off the amount of bunk space available. In order to achieve the desired manger space allocation, red marks were painted on the concrete feed bunk to identify the targeted feed delivery area. A total of 203 cm for the 8IN treatment and 406 cm for the 16IN treatment was required for the targeted delivery area out of the 762 cm of bunk space available. Additionally, heated concerted waterers (Bohlmann Quality Products; Denison, IA) were split between adjacent pens and were 78.74 cm × 55.88 cm × 60.96 cm in dimension and occupied approximately 0.22 m^2^ of pen space.

### Dietary Management

Orts were collected, weighed, and dried in a forced air oven at 100 °C for 24 h to determine DM content if carryover feed spoiled, or was present on weigh days. If carryover feed was present on weigh days, the residual feed was removed prior to the collection of BW measurements. The DMI of each pen was adjusted to reflect the total DM delivered to each pen after subtracting the quantity of dry orts for each interim period. Actual diet formulation and composition were based upon weekly DM analyses (drying at 60 °C until no weight change), tabular nutrient values (Preston, 2016), and corresponding feed batching records. Weekly DM determination (method no. 935.29) was used to determine the DM content of each ingredient fed each week (AOAC, 2012, 2016).

Fresh feed was manufactured twice daily in a stationary mixer (2.35 m^3^; readability 0.454 kg). Diets (Table 1; DM basis) consisted of ingredients common to the northern plains feeding region and changed over time because of evolving ingredient inventory. Liquid supplement was included to provide monensin sodium (Rumensin 90; Elanco, Indianapolis, IN) at 30 g/907 kg (DM basis) and vitamins and trace minerals to meet nutrient requirements for growing and finishing beef cattle (NASEM, 2016). A type B Melengestrol acetate (MGA, Zoetis) product (1 mg/0.45 kg) was manufactured and included at a rate of 0.225 kg/heifer daily in replacement of dry-rolled corn to suppress heifer cyclicity. Diets presented in Table 1 are actual DM diet composition, plus tabular nutrient concentrations and tabular energy values (Preston, 2016).

**Table 1.** Actual dietary formulation and tabular nutrient content for heifers offered a limit fed diet and 20.3 (8IN) or 40.6 (16IN) cm of linear bunk space per heifer through the 109 d feeding experiment^1^

Item	Day 1 to 22	Day 23 to 82	Day 83 to 109
Dry-rolled corn^2^, %	20.80	16.42	48.04
High-moisture corn, %	43.23	33.40	–
Dried distillers grains plus solubles, %	14.71	15.28	15.01
Oat hay, %	15.36	–	–
Corn silage, %	–	29.82	32.03
Liquid supplement^3^, %	5.89	5.08	4.92
Diet dry matter, %	79.65	57.04	54.68
Crude protein, %	13.31	12.94	12.80
Neutral detergent fiber, %	21.02	24.16	24.91
Acid detergent fiber, %	11.13	13.21	13.69
Ash, %	6.46	6.05	6.03
Organic matter, %	93.54	93.95	93.97
Ether extract, %	3.59	3.59	3.58
Tabular net energy for maintenance, Mcal/kg	2.05	2.00	1.97
Tabular net energy for gain, Mcal/kg	1.36	1.35	1.32

^1^All values except DM on a DM basis.

^2^Melengestrol acetate (MGS, Zoetis) was included at 0.50 mg/heifer daily in a premix that replaced a portion of the dry-rolled corn.

^3^From d 1 to 22 (dry-matter basis): 36.27% crude protein, 28.00% non-protein nitrogen, 1.63 Mcal/kg net energy for maintenance, 1.10 Mcal/kg net energy for gain, 1.62% crude fat, 4.62% Ca, 0.43% P, 2.28% K, 0.47% Mg, 5.00% salt, 3.38% Na, 0.54% S, 4.00 ppm Co, 200.00 ppm Cu, 20 ppm I, 25.15 mg/kg EDDI, 150.29 ppm Fe, 400.00 ppm Mn, 3.08 ppm Se, 700.00 ppm Zn, 44,092 IU/kg Vitamin A, 440.92 IU/kg Vitamin E, Monensin 500 g/907-kg. From d 23 to 109 (dry-matter basis): 41.86% crude protein, 38.38% non-protein nitrogen, 0.95 Mcal/kg net energy for maintenance, 0.66 Mcal/kg net energy for gain, 0.91% crude fat, 10.89% Ca, 0.32% P, 7.00% K, 0.22% Mg, 6.03% salt, 3.07% Na, 0.33% S, 4.23 ppm Co, 199.88 ppm Cu, 11.99 ppm I, 15.07 mg/kg EDDI, 83.16 ppm Fe, 304.81 ppm Mn, 2.90 ppm Se, 664.59 ppm Zn, 44,064.55 IU/kg Vitamin A, 376.52 IU/kg Vitamin E, Monensin 579.35 g/907-kg

### Growth Performance Calculations

Heifers were individually weighed at study initiation and on d 14, 35, 63, 84, and 109 (final day of the experiment). Cumulative daily weight gain was based upon initial shrunk BW (4% shrink) and final shrunk BW (2% shrink). Average daily gain (ADG) was calculated by subtracting the final shrunk BW from the initial shrunk BW and dividing by days on feed. Gain to feed ratio (G:F) was calculated by dividing ADG by DMI.

### Dietary NE Utilization Calculations

Observed dietary NE was calculated from daily energy gain (EG; Mcal/d) according to the medium frame steer calf equation using mean equivalent BW [median feeding BW × (478/534)]: EG, Mcal/d = ADG^1.097^ × 0.0557W^0.75^; EG was the daily deposited energy and W was the mean equivalent BW (NASEM, 2016). Maintenance energy required (EM; Mcal/d) was calculated by the following equation: EM, Mcal/d = 0.077BW^0.75^ (Lofgreen and Garrett, 1968; NASEM, 2016) where BW was the average of initial shrunk BW and final shrunk BW (initial BW shrunk 4% and final BW shrunk 2%). Using the estimates required for maintenance and gain, the observed dietary NEm and NEg values of the diet were generated using the quadratic formula: $x=\frac{-b\pm \sqrt{b^{2}-4ac}}{2c}$, where x = NE_m_, Mcal/kg, a = −0.41EM, b = 0.877EM + 0.41DMI + EG, c = −0.877DMI, and NE_g_ was determined from: 0.877 NE_m_ – 0.41 (Zinn and Shen, 1998; Zinn et al., 2008a). The ratio of observed-to-expected NE ratio was determined from observed dietary NE for maintenance or gain divided by tabular NE for maintenance or gain.

To calculate the daily DMI required, the following equation was used: DMI (kg) = FFM + FFG. Feed for maintenance (FFM; kg) was the EM divided by the tabular NEm value. Feed for gain (FFG; kg) was the RE was divided by the tabular NEg value. The following equation was used to determine retained energy (RE; Mcal) = 0.0557 × EQSBW^0.75^ × SWG^1.097^. Equivalent shrunk BW (EQSBW; kg) = [(current shrunk BW + target shrunk BW of the next period)/2] × (standard reference weight of 478 kg divided by the mature shrunk BW, which was assumed to be 575 kg based on similar calves from previous research). Shrunk weight gain (SWG) was targeted to be 1.36 kg/d.

To calculate predictive values, a final BW of 575 kg was assumed to be the mature BW of the heifers in the study. This was determined off previous data on steers of similar composition with a mature BW of 625 kg (Smith, 2020). An adjustment of 50 kg was assumed as heifers finish at a lower mature BW compared to steers of similar composition (Zinn et al., 2008b)

### Statistical Analysis

Data were analyzed using analysis of variance appropriate for a randomized complete block design experiment using the GLIMMIX procedures of SAS 9.4 (SAS Inst. Inc. Cary, NC). Manger space allocation was included as a fixed effect, and block was considered a random factor; pen served as the experimental unit for all analyses. Least squares means (LSMEANS) were generated and treatment effects were analyzed and separated least significance differences using the PDIFF with LINES option. PDIFF requests P-values for differences of the least squares means (as a matrix of pairwise values) and LINES uses connecting lines to indicate insignificant subsets of least squares means. An α of 0.05 determined significance and an α of 0.06 to 0.10 was considered a tendency.

## Results and Discussion

As previously mentioned, feed intake management is not a new concept to the cattle feeding industry (Galyean et al., 1999). This methodology is most commonly used in mature beef cows to avoid over-feeding and accumulating excessive body conditions. However, the CNES has been shown to be an effective means to calculate the predictive rate of gain measures in feedlot cattle (NASEM, 2016). Galyean et al (1999) suggested that the CNES equations are based on using a 4% shrink in the equations. In the present study, a 2% shrink was used to shrink the final BW (after 109 d of limit-feeding) to more accurately account for gastrointestinal tract fill, as the heifers were limit-fed. When a 2% shrink was applied, our observed gains were in good agreement with predicted values. When Zinn (1989) used the same equations, observed and predicted gains were in close agreement, 4.1% higher than expected (1.45 kg/d) and 1.6% lower than expected (1.22 kg/d) for two different experiments. When steers were programmed to gain 1.35 or 1.5 kg daily (Hicks et al., 1990), observed daily gains were 13% and 17% lower than calculated, respectively. In the present study, observed daily gains were 4.3% and 2.7% less than targeted expectations for 8IN and 16IN, respectively (Table 2).

**Table 2.** Cumulative growth performance responses for heifers offered a limit-fed diet and 20.3 (8IN) or 40.6 (16IN) cm of linear bunk space per heifer through the 109 d feeding experiment^1^

	Bunk space treatment (centimeters/heifer)			
Item	8IN (20.3 cm)	16IN (40.6 cm)	SEM	*P*-value
Pens, *n*	5	5	–	–
Heifers, *n*	50	49	–	–
**Cumulative growth**				
Initial BW, kg	329	329	0.59	0.77
Final BW, kg	471	474	2.95	0.43
ADG, kg	1.30	1.32	0.027	0.46
DMI, kg	8.04	8.04	0.002	0.35
G:F	0.16	0.17	0.003	0.46
S.D. ADG	0.40	0.36	0.039	0.39
**Applied energetics measures** ^2^				
Observed net energy for maintenance (NEm), Mcal/kg	1.99	2.01	0.027	0.44
Observed net energy for gain (NEg), Mcal/kg	1.34	1.35	0.024	0.44
Observed to expected NEm	1.00	1.01	0.013	0.47
Observed to expected NEg	0.98	0.99	0.016	0.42

^1^A 4% shrink was applied to the initial BW measure to account for digestive tract fill and all subsequent BW measures were pencil shrunk 2% to account for digestive tract fill.

^2^Calculated from observed cumulative growth performance assuming a mature BW of 534 kg and a tabular NEm and NEg of 2.00 Mcal/kg and 1.34 Mcal/kg of NEm and NEg, respectively.

There were no differences (*P* > 0.35; Table 2) observed between the 8IN or 16IN heifers for initial BW, final BW, ADG, DMI, G:F, or applied energetic measures, nor was the S.D. of ADG influenced by manger space allowance (*P* = 0.39). Our observations agree with other published results where restricting bunk access had no effect on the performance of program-fed cattle (Gunter et al., 1996; Zinn, 1989). These data suggest that using tabular NE values and required NEm and RE equations are an effective means to program cattle to gain a desired rate of gain per day, independent of bunk space allowance. These results from the present study also confirm that manger space allotments greater than 15 cm of linear bunk space per head do not appreciably enhance feedlot growth performance, as was suggested by Zinn (1989).

Harrison and Oltjen (2021) reported decreased ADG with a greater coefficient of variation when bunk restrictions were imposed on steers fed a finishing diet. A potential explanation for the greater variation observed in some experiments is increased competition between animals for feed (Longenbach et al., 1999). Behavioral differences were not specifically measured in the current experiment; however, heifers in the 8IN treatment did appear to display greater agonistic behavior while adapting to bunk space limitations. Targeted observations of cattle behavior during adaptation to program feeding could provide valuable insight to better understand the effect of social structure on diet adaptation.

It has been suggested that programmed and restricted feeding can be used as a method to help identify sick cattle (Harrison and Oltjen, 2021). There were no differences in health (*P* > 0.05; data not reported) observed in the present study and no cattle showed signs of morbidity. However, this method to identify sick cattle seemed to be only effective in smaller-scale pens with severe restriction (Galyean et al., 1999). This technique would require further evaluation in higher-risk cattle with more severe restrictions and in a more practical setting to be confirmed. Although not statistically analyzed, 8IN heifers appeared to have looser stools during the first two weeks of the study compared to the 16IN heifers. At this time, there is no literature that has observed differences in stools based on manger space restriction and/ or limit feeding.

## Implications

Restricting manger space allocation from 40.6 to 20.3 cm did not negatively influence gain efficiency or the efficiency of dietary NE utilization in heifers program fed a concentrate-based diet to gain 1.36 kg daily. Current estimates for maintenance and RE can be applied to heifers if mature BW is known. Additionally, tabular ingredient values from current feeding standards work well under Northern Plains feedlot conditions. Overall, the equations set forth by the CNES prove to be an efficacious method for managing gain and feed intake. This may help to provide a strategic method for managing feed ingredient inventory. 

## Data Availability

Data can be made avaialable with reasonble request to Z.K.S.
